# Self-reported sleep relates to microstructural hippocampal decline in ß-amyloid positive Adults beyond genetic risk

**DOI:** 10.1093/sleep/zsab110

**Published:** 2021-04-28

**Authors:** Håkon Grydeland, Donatas Sederevičius, Yunpeng Wang, David Bartrés-Faz, Lars Bertram, Valerija Dobricic, Sandra Düzel, Klaus P Ebmeier, Ulman Lindenberger, Lars Nyberg, Sara Pudas, Claire E Sexton, Cristina Solé-Padullés, Øystein Sørensen, Kristine B Walhovd, Anders M Fjell

**Affiliations:** 1 Research Group for Lifespan Changes in Brain and Cognition, Department of Psychology, University of Oslo, Oslo, Norway; 2 Department of Radiology and Nuclear Medicine, University of Oslo, Oslo, Norway; 3 Departament de Medicina, Facultat de Medicina i Ciències de la Salut, Universitat de Barcelona, Barcelona, Spain; 4 Lübeck Interdisciplinary Platform for Genome Analytics (LIGA), Institutes of Neurogenetics and Cardiogenetics, University of Lübeck, Lübeck, Germany; 5 Center for Lifespan Psychology, Max Planck Institute for Human Development, Berlin, Germany; 6 Department of Psychiatry, University of Oxford, Oxford, UK; 7 Max Planck UCL Centre for Computational Psychiatry and Ageing Research, Berlin, Germany, and London, UK; 8 Umeå Center for Functional Brain Imaging, Umeå University, Umeå, Sweden

**Keywords:** hippocampus, sleep, β-amyloid, Alzheimer’s disease, memory, aging, lifespan, longitudinal, mean diffusivity

## Abstract

**Study Objectives:**

A critical role linking sleep with memory decay and β-amyloid (Aβ) accumulation, two markers of Alzheimer’s disease (AD) pathology, may be played by hippocampal integrity. We tested the hypotheses that worse self-reported sleep relates to decline in memory and intra-hippocampal microstructure, including in the presence of Aβ.

**Methods:**

Two-hundred and forty-three cognitively healthy participants, aged 19–81 years, completed the Pittsburgh Sleep Quality Index once, and two diffusion tensor imaging sessions, on average 3 years apart, allowing measures of decline in intra-hippocampal microstructure as indexed by increased mean diffusivity. We measured memory decay at each imaging session using verbal delayed recall. One session of positron emission tomography, in 108 participants above 44 years of age, yielded 23 Aβ positive. Genotyping enabled control for *APOE* ε4 status, and polygenic scores for sleep and AD, respectively.

**Results:**

Worse global sleep quality and sleep efficiency related to more rapid reduction of hippocampal microstructure over time. Focusing on efficiency (the percentage of time in bed at night spent asleep), the relation was stronger in presence of Aβ accumulation, and hippocampal integrity decline mediated the relation with memory decay. The results were not explained by genetic risk for sleep efficiency or AD.

**Conclusions:**

Worse sleep efficiency related to decline in hippocampal microstructure, especially in the presence of Aβ accumulation, and Aβ might link poor sleep and memory decay. As genetic risk did not account for the associations, poor sleep efficiency might constitute a risk marker for AD, although the driving causal mechanisms remain unknown.

**Significance Statement**

Sleep links with memory decay and β-amyloid (Aβ) accumulation, two markers of Alzheimer’s disease (AD) pathology, and a critical role connecting these factors may be played by hippocampal integrity. We performed a longitudinal study testing how self-reported sleep patterns related to changes in memory and intra-hippocampal microstructure, including in the presence of Aβ. We found that worse global sleep quality and sleep efficiency related to more rapid reduction in hippocampal microstructure. In older adults, the relation was stronger in presence of Aβ accumulation. Sleep efficiency related to memory decay indirectly via hippocampal decline. Genetic risk for sleep efficiency and AD did not explain the results, which suggest that poor self-reported sleep efficiency might constitute a separate risk marker for AD.

## Introduction

Individuals with sleep disturbances have increased risk for Alzheimer’s disease (AD) [1], and accumulation of β-amyloid (Aβ) [2, 3]. Aβ is modestly related to memory decline [4], and studies have suggested that relations between Aβ and memory partly depend on sleep [5, 6]. A critical role in linking sleep to Aβ and memory may be played by hippocampal integrity. Hippocampal damage has been related to disturbed sleep processes as measured by electrophysiology [7], and we have previously shown that worse self-reported sleep related modestly to hippocampal atrophy across samples [8]. Macrostructural atrophy likely reflects long-term effects of microstructural decline such as dendritic spine density reduction [9], which in mice has been detected in the hippocampus after sleep deprivation [10]. Integrity measured by diffusion tensor imaging (DTI) may detect subtle microstructural decline [11]. DTI-derived diffusivity measures such as mean diffusivity (MD) have proven sensitive to age-related lifespan differences [12, 13] and changes [14], indicating that decline in the parahippocampal cortex might begin already in the third decade of life [13]. MD measured in the hippocampus has demonstrated sensitivity to early AD abnormalities [15], and has been linked to memory [16], including in a longitudinal sample covering almost the entire lifespan [17]. We therefore hypothesized that hippocampal integrity as measured by MD would be sensitive to variation in subjective sleep quality.

Sleep-hippocampal integrity relationships could reflect effects of the *APOE* ε4 genotype [18], the genetic variant most strongly related with AD, or of common genetic variation affecting sleep and hippocampus [19]. While recent large scale genomics studies have identified overlapping genomic regions associated with sleep-related traits [20] and risk for AD [21], the effect sizes per genetic variant are too small to allow for detailed mechanistic studies. By using polygenic scores (PGSs) [22], we can measure the overall genetic risk, tagged by common genetic variants, of sleep problems and AD, respectively. Taken together, testing whether worse self-reported sleep relates to memory decline and more rapid reduction of hippocampal integrity while controlling for genetic variation in both sleep and AD, and whether such relations are stronger in older adults with pathological levels of Aβ, might aid in the quest to decipher the role of sleep problems in early AD-related pathology.

Here, in 243 cognitively healthy participants aged 19–81 years, we asked whether self-reported sleep characteristics were associated with decline in memory and microstructural (MD) hippocampal integrity over an average of 3 years. In a previous study of hippocampal volume loss [8], we found that relationships with sleep problems did not vary with age, suggesting that sleep-hippocampus relations are best studied by including the entire adult lifespan. We hypothesized that worse sleep would relate to stronger decline, particularly in individuals with cortical Aβ accumulation. As better sleep has been shown to have attenuating effects of the *APOE* ε4 genotype on AD risk [23], and sleep–atrophy relations have been found to be partly independent of *APOE* genotype [24], we hypothesized the relations to remain when controlling for *APOE* ε4 status and PGSs for sleep and AD, respectively. Finally, we have previously linked self-reported sleep with hippocampal atrophy [8] using data from the Lifebrain consortium [25]. For the current analyses, we did not have access to longitudinal DTI data from the consortium, but did have the opportunity to further probe sleep relations with memory decline to increase generalizability of the results, by performing a meta-analysis using one-time point self-reported sleep reports and memory change scores.

## Methods

### Sample

The sample was drawn from projects consisting of 2–6 study waves at the Center for Lifespan Changes in Brain and Cognition, Department of Psychology, University of Oslo, Norway. The Regional Ethical Committee of Southern Norway approved all procedures, and all participants consented in writing prior to commencement. At baseline, participants were recruited through advertisements. At follow-up, recruitment was by written invitation to the original participants. At both baseline and follow-up, participants underwent health interviews, and were required to be right-handed, fluent Norwegian speakers, and have normal or corrected to normal vision and hearing. Exclusion criteria were history of injury, disease or psychoactive drug use affecting central nervous system function, including clinically significant stroke, serious head injury, untreated hypertension, and diabetes, as well as MRI contraindications. Based the availability of a completed sleep questionnaire, and valid baseline and follow-up anatomical MRI and DTI scans, 251 community-dwelling participants were eligible for inclusion (see Figure S1 for attrition of participants). Additional inclusion criteria for the present analyses were (1) valid scores at baseline and follow-up on the long delay free recall of the California Verbal Learning Test (CVLT, see below for details, seven participants lacked scores at follow-up), and (2) as in our previous work [26], CVLT long delay free recall change between visits <60% (one participant excluded), in line with our previous work on sleep and hippocampus [26]. The final sample consisted of 243 participants (62% female, mean baseline age = 54, range: 19–81, see Table 1 for details, and Figure S2 for the age distribution).

**Table 1.** Participants demographics

				Correlation	
	*M*	*SD*	Range	*PSQIg*	*Age*
**Age, baseline (females = 62%)**	53.7	19.9	19–81	.12*	NA
**Sleep**					
**Global**	5.0	2.8	0–14	NA	.12*
**Quality**	0.8	0.7	0–3	.75**	.04
**Latency**	1.0	0.8	0–3	.73**	−.08
**Duration**	0.6	0.7	0–3	.60**	.14*
**Efficiency**	0.5	0.8	0–3	.66**	.21**
**Disturbance**	1.1	0.5	0–2	.48**	.17*
**Daytime dysfunction**	0.7	0.6	0–2	.30**	−.27**
**CVLT, 30-min delayed recall (SPC)**	0.3	9.8	–40–39	−.14*	−.18**
**Interval MRI**	3.1	1.2	1–6	−.07	−.45**
**Head movement (tSNR), baseline**	5.9	0.6	4–7	−.03	−.63**
**Head movement (tSNR), change**	0.1	0.5	–1–2	.01	.16*
**Interval PSQI to baseline MRI (years** ^**a**^)	0.6	0.8	–1–4	.08	.04
**Interval PSQI to follow-up MRI (years** ^**a**^)	−2.5	1	–5−0	−.12	−.52**
**Interval PET scan to follow-up MRI (years)**	−1.0	0.9	−2−1	−.04	−.06

Abbreviations: NA, not applicable; PSQI, Pittsburgh Sleep Quality Inventory; PSQIg, PSQI global score; tSNR, temporal signal to noise ratio; SPC, symmetrized percent change.

**p < .001; *p < .05.

^a^Missing exact date for 14%.

Participants had full-scale IQ above 85 on the Wechsler Abbreviated Scale of Intelligence [27], except two participants aged 64 and 27 years, scoring 79 and 83 at baseline (both scored > 85 on follow-up). On the Mini Mental State Examination (MMS) [28], participants above 40 years of age scored ≥26, except two participants aged 80 years scoring 25. All participants who completed the Beck Depression Inventory (BDI) scored ≤16, except four participants, aged 24–45 at follow-up, scoring 18–24. Eighty-one participants aged above 68 years completed the Geriatric Depression Scale (GDS) [29], and all scored ≤9 except for seven participants (five participants aged 71–74 at follow-up, and two participants aged 73 and 77 years at baseline who scored at non-depression levels on follow-up). A depression score was missing for 15 participants, either at one time point (13 participants, aged 19–77 years, all scoring ≤ 7 on BDI) or both (two participants, aged 29 and 58 years). To account for potential influences of particularly depression, we undertook sensitivity analyses (see below). A neuroradiologist evaluated the MRI scans, and all participants were deemed free of significant injuries or pathological conditions.

**Figure 1.**
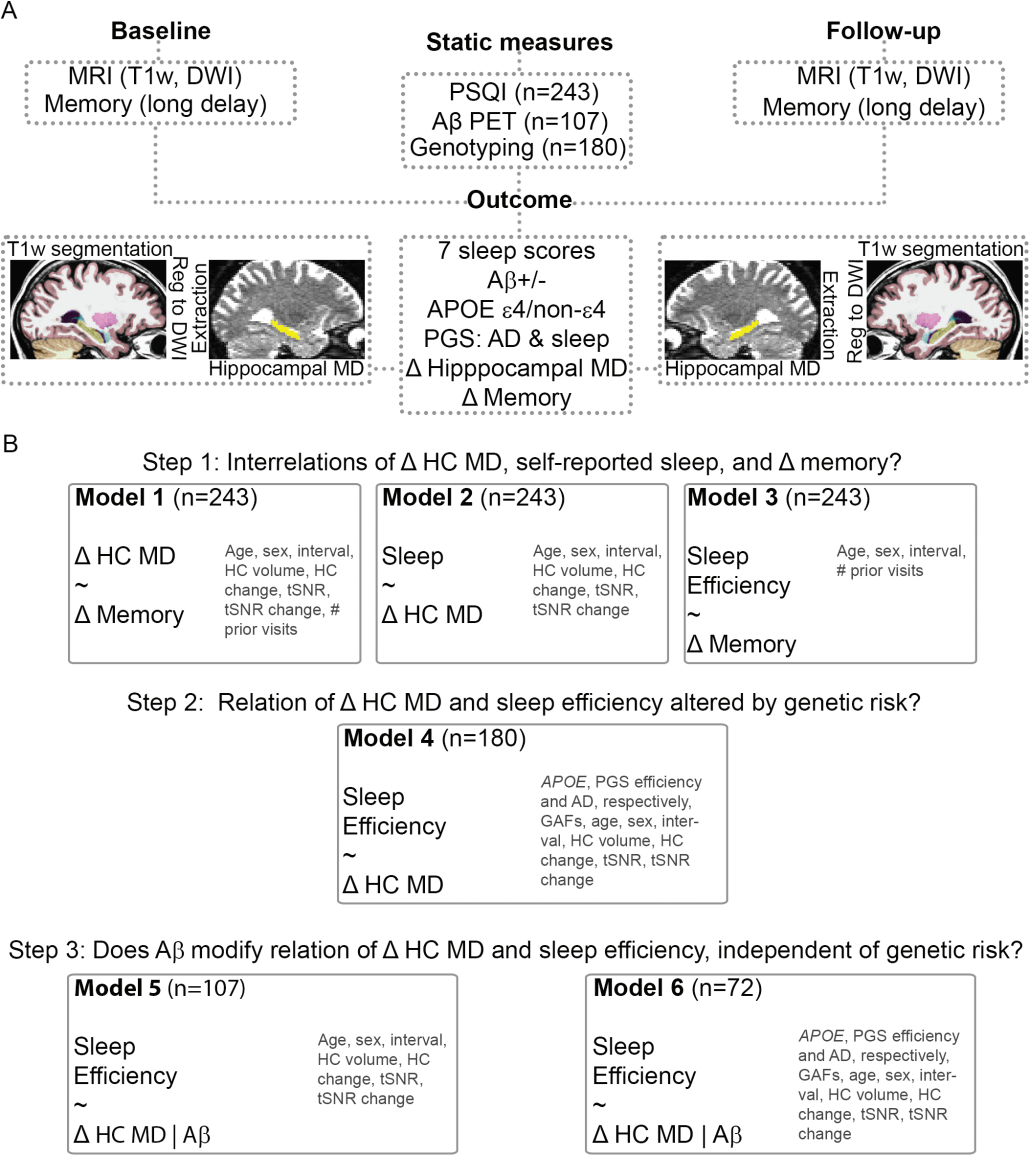
Study overview. (A) Study design. (B) Main regression models. Covariates are named in dark gray font color. Abbreviations: Age, baseline MRI age; HC, hippocampus; HC volume, baseline hippocampal volume; tSNR, temporal signal to noise ratio, derived from DWI scans (see text for details); PSQI, Pittsburgh Sleep Quality Inventory; Aβ, β-amyloid; PGS, polygenic scores; GAF, genetic ancestry factor; # prior visits, number of prior visits.

Figure 1, A shows the study design. Similar to our previous work on self-reported sleep [26], baseline MRI was administered between 2011 and 2016, and follow-up MRI between 2015 and 2018. PSQI was completed once by each participant, between 2012 and 2017, on average 0.6 (*SD* = 0.8) years after baseline MRI (16 participants completed the PSQI on average 0.4 (*SD* = 0.3) years before baseline MRI, while exact completion date was not available for 34 participants). As shown in Table 1, the PSQI to follow-up MRI interval was correlated with age, as participants aged 70–80 had a shorter interval than the majority of the sample, except for a group of young adults, allowing modelling of potential confounding effects (age was used as covariate of no interest in all analyses). The memory assessments were performed on average 13 (*SD* = 22) days before the baseline MRI, and 26 (*SD* = 29) days before the follow-up MRI, respectively. PET scanning was performed once in a subset of participants, between 2015 and 2018, on average 1 (*SD* = 0.9) year before the MRI follow-up.

### Sleep assessment

To assess sleep, we used the Pittsburgh Sleep Quality Index (PSQI) [30]. This self-report index yields one global sleep quality score, which is the sum of the score of seven components: (1) quality, (2) latency, (3) duration, (4) habitual efficiency, (5) disturbance, (6) use of sleep medication, and (7) daytime dysfunction. We tested relations between hippocampal integrity and the global sleep quality score and all components, except the sixth component as use of medication was an exclusion criterion. Components on the original ordinal scale were chosen over individual questions or components as continuous variables to (1) enable evaluation of all sleep characteristics from the PSQI while simultaneously limiting the number of items to test for increased power, and (2) to not unduly bias the analyses toward the majority of participants who showed good sleep (for instance, 83% of the sample had sleep efficiency scores above 80%). In PSQI, efficiency is calculated as sleep duration (hours slept) divided by the number of hours spent in bed, times 100, and then given score of 0–3 for >85%, 75–84%, 65–74%, and <65%, respectively. Although the PSQI asks about sleep patterns of the last month, here, as in our previous longitudinal work [26], we take the PSQI to reflect relatively stable sleep patterns, an inference for which there is support in adults above 38 years [31, 32] and in a recent finding showing that baseline and follow-up PSQI did not change significantly over a 3-year interval, and scores between the time points were correlated (*r* = .81, *p* < 10^−14^) [33].

### MRI acquisition

Diffusion tensor imaging scans were acquired at two Siemens scanners (Siemens Medical Solutions, Erlangen, Germany), a 1.5 T Avanto (*n* = 64, 70% female, mean age (*SD*, min–max) = 51 (13, 24–77) years), TR/TE = 8,200/81 ms, FOV = 128, 60 diffusion-sensitizing gradients at a *b*-value of 700 s mm^−2^ and two volumes without diffusion weighting (*b*-value = 0), and 3 T Skyra scanner (*n* = 187, 58% female, mean age (*SD*, min–max) = 55 (22, 19–81) years), TR/TE = 9,200/87 ms, FOV = 130, 64 diffusion-sensitizing gradients at a b-value of 1,000 s mm^−2^ and 1 volume without diffusion weighting. The sequences and scanner were the same across the two time points for each participant.

### Preprocessing

The diffusion-weighted data were analyzed using the FMRIB Software Library (see SI for details), and included susceptibility-induced field correction, and correction for head motion, signal dropout, and eddy current-induced fields [34]. After removing nonbrain tissue, and estimating the diffusion ellipsoid properties (the length of the longest, middle, and shortest axes, called eigenvalues) in each voxel, we computed mean diffusivity (MD), defined as the mean of the three eigenvalues. Thus, MD reflects the average degree of water molecule diffusion. We employed a DTI-derived measure as results indicate that DTI can detect subtle effects in microstructure, including age-related lifespan differences [12, 13] and changes [14]. The latter study showed how longitudinal DTI provides sensible results in the white matter. In grey matter, the coherence in fiber direction, and hence water diffusion, is lower compared with the white matter, and MD is arguably the preferable DTI index [13]. MD measured in the hippocampus has shown sensitive to very early AD [15], and to memory [16, 35]. In a recent longitudinal effort, hippocampal MD showed relations with memory across almost the entire lifespan (4–93 years of age, 3,367 scans, and 3,033 memory test sessions over 1–6 time points, spanning an interval up to 11.1 years) [17]. Longitudinal tissue changes in human hippocampal MD have also shown to be reliable across cohorts, and have been validated by similar findings in rats [36].

### Hippocampus segmentation and DTI registration

The T1-weighted image was automatically processed with FreeSurfer software suite (version 6.0.0), independently for each time point, yielding segmentation of left and right hippocampus [37]. To extract MD from the hippocampi in native DTI space for each participant, a *B* = 0 volume from the diffusion data was registered to the T1-weighted image in FreeSurfer space with a within-subject, cross-modal registration using a boundary-based cost function constrained to be 6 degrees of freedom [38]. The resulting registration matrix was inverted, and applied to the segmentation of the left and right hippocampus, yielding hippocampus masks in native diffusion space. The masks were binarized using *mri_binarize* at a minimum voxel threshold of 1, for the most restricted masks compared with lower thresholds. To reduce the number of tests, we calculated the average hippocampal MD based on the left and right hippocampus at each time point.

### Memory change

The participants underwent neuropsychological testing including memory assessment via the CVLT. This CVLT test was administered following the standard procedure, with a list of 16 words read to the participant over five trials. After each trial, the participant was asked to repeat all of the words she/he could remember. Following the five trials, an interference list was read, and the participant was asked to repeat all of the words she/he could remember from the new list. The interference list was followed by a short delay free recall of the first list. Approximately 20–30 min later, there was a *long delay free recall* of the first list. In an effort to minimize practice effects due to repeated testing, we administered different Norwegian versions of the CVLT, including the CVLT I, CVLT II original and alternate version [39–41], in different follow-up waves of the lifespan sample. We chose the arguably most sensitive measure of hippocampus-dependent memory, namely *long delay free recall*, that is, the number of correctly recalled words after an approximately 30-minute delay.

### Symmetrized percent change (SPC)

As in our previous longitudinal sleep work [26], we calculated symmetrized percent change (SPC), as symmetrized measures have been shown to be more robust, and with equal or greater statistical power [42]. For the average hippocampus value at baseline and follow-up (AH1 and AH2), the SPC was obtained by the following formula: SPC = 100 * (AH2 − AH1)/(AH2 + AH1). The same formula was used to obtain SPC measure for memory change.

### PET acquisition

A total of 108 participants (mean age (*SD*, min–max) = 68.0 (8.7, 44.4–80.8) years) underwent [18] F-flutemetamol-PET scan, sensitive to Aβ accumulation. Images were acquired on a General Electric Discovery PET/CT 690 scanner at Aleris Hospital and Radiology, Oslo, Norway. A low-dose computerized tomography scan was first performed for subsequent attenuation correction of the PET scan. Participants were injected with 200 ± 20 MBq [18] F-flutemetamol as a bolus and examined 90 min later. Three-dimensional dynamic data were acquired in list mode for 20 min, with the following parameters: 47 image planes, voxel size = 1.33 mm × 1.33 mm × 3.27 mm, field of view = 256 mm. The images were reconstructed using the VUEPoint HD Sharp iterative reconstruction algorithm. This algorithm adds resolution recovery in an iterative reconstruction loop by incorporating information about the PET detector response which improves resolution and contrast recovery compared with traditional analytic methods [43]. We used four iterations, 16 subsets, time of flight, and a full width at half maximum Gaussian post-filter of 3 mm. As we were interested in the gross tracer uptake, we binned the data into a single frame, and submitted this static PET image to further pre-processing and value extraction.

### Genetic data

A subsample of 179 participants (64% females, mean age (*SD*, min–max) = 53.7 (20.4, 20.1–80.8) years had genome-wide single nucleotide polymorphisms (SNPs) and manual *APOE* ε4 genotypes available. Buccal swab and saliva samples were collected for DNA extraction followed by genome-wide genotyping using the “Global Screening Array” (Illumina, Inc.). A*POE* ε4 (rs429358) status was determined using TaqMan (Thermo Fisher Scientific, Inc.) chemistry. Detailed information on DNA collection, quality control, genotyping, and imputation has been reported elsewhere [44]. The PGSs of sleep efficiency and AD were computed using summary statistics from previously published genome-wide association studies (GWAS) [21, 45]. These statistics were based on SNPs with *p*-values <.01 in the respective GWAS, except for variants located in the extended MHC region (build hg19; chr6:25,652,429–33,368,333), where we included the most significant SNP. After removing the *APOE* gene region (build hg19; chr19:44,909,011–45,462,650) for which we used the manually derived ε4 (rs429358) genotypes instead, we used the software PLINK [46] to implement the following steps: (1) clumping of the GWAS summary statistics by the –clump option with parameters –clump-p1 1.0 –clump-p2 1.0 –clump-kb 500 –clump-r2 0.1. The linkage disequilibrium (LD) structure was based on the European subpopulation from the 1,000 Genomes Project Phase3 [47]. (2) Deriving PGSs for our sample using the –score function. To control for population substructures, we computed the genetic ancestry factors using principal component methods [48], and included only participants of European ancestry in the genetic subsample analysis. The PGS for sleep efficiency was based on a genome-wide association study using accelerometer-derived mean sleep efficiency (calculated as proportion of sleep period time-window classified as sleep) [45], and in our sample a higher PGS reflected a higher genetic propensity towards more efficient sleep. The AD PGS was based on a genome-wide meta-analysis of clinically diagnosed AD and AD-by-proxy (based on parental diagnoses) [21], and in our sample a higher PGS reflected a higher AD risk. To test for the effect of *APOE* separately from the common genetic variation reflected by the polygenic scores, we estimated *APOE* ε4 counts by determining the haplotypes of the two SNPs rs7412 and rs429358 [49, 50], coded as 0, 1, or 2 copies of the ε4 allele, and binarized to ε4-non-carrier or ε4-carrier.

### PET pre-processing

We used *PetSurfer*, a set of tools within the FreeSurfer suite, for partial volume correction [51]. Specifically, for each participant, we registered the static PET image to the anatomical T1-weighted image using boundary-based registration [38]. This registration was inverted to get a high-resolution segmentation (upsample factor = 2) from the high-resolution MRI space in PET space, and simultaneously perform the partial volume correction with the Symmetric Geometric Transfer Matrix method, as recommended when using regions of interests (instead of vertex-wise) approach [51, 52]. This procedure yielded PET signals for each of the 68 cortical regions in Desikan-Killiany atlas [53]. We used cortical regions as Aβ has been reported to appear first in cortex [54]. The PET signal in each cortical region was divided by the mean signal of the cerebellum cortex to obtain standardized uptake value ratios (SUVR) [55].

### Aβ status

As common in the literature [55], we dichotomized the SUVR into high or low Aβ groups using a data-driven approach. We ran a principal component analysis on SUVR from the 68 cortical regions using the *prcomp* function (R package *stats* v3.6.1 [56], values were zero-centered and scaled to have unit variance), and extracted the first component (which explained 66.7% of the variance, while, for comparison, the second component explained 7%). The cut-off between groups was determined using Gaussian mixture modeling (R package *mclust* v5.2 [57]). We fitted 18 models, ranging from 1 to 9 mixtures, allowing for either equal or unequal variance, and selected the model with the lowest Bayesian information criterion value. As previously reported in healthy older participants [55], the optimal model consisted of a two-distribution model with unequal variance. Participants with a >.5 probability of belonging to the high Aβ distribution were classified as *Aβ positive*, and the remaining as *Aβ negative*.

### Meta-analysis of self-reported sleep and memory change

To further assess the relation between sleep and memory change, we performed a meta-analysis sleep and memory data from the Lifebrain consortium (https://www.lifebrain.uio.no/) [25], an EU-funded (H2020) project including participants from several major European brain studies: Berlin Study of Aging-II (BASE-II) [58, 59], the BETULA project [60], University of Barcelona brain studies [61–63], and Whitehall-II [64], yielding a total of 1,196 participants. The samples and procedures used are described in detail elsewhere [65]. The data available in all projects were (1) self-reported sleep scores from one time point, and (2) memory change score between two time points. Sex, age, and interval between memory tests were entered as covariates. All subsamples used the PSQI for sleep evaluation, except the Betula sample, which used the Karolinska Sleep Inventory (for details of conversion to PSQI scores, see [65]). The following memory tests were used: 30-min delayed free recall from the Verbal Learning and Memory Test (BASE-II), an immediate free recall of sentences (Betula), 30-min delayed recall from the Rey Auditory Verbal Learning Test (Barcelona), a short-term 20 word free recall test (Whitehall-II) [66].

### Study design and statistical analysis

Our main question of a relation between sleep and microstructural hippocampus change was addressed by multiple regression models testing seven PSQI variables vs. hippocampal MD change (see Figure 1, B for main regression models). To correct for the multiple tests, we adjusted the seven resulting p-values by applying false discovery rate (FDR) correction (*p.adjust* function, R *stats* version 3.6.1). Head movement is a potential important confound in brain imaging studies. As a proxy measure of head movement during scanning, we calculated temporal signal-to-noise ratio from the diffusion scans [67], which increased with age (*R*^2^=.40, *p* < .001). We included this ratio in all hippocampal MD analyses as covariate of no interest, in addition to hippocampal volume at baseline MRI, and difference in movement and hippocampal volume between baseline and follow-up MRI. The latter two covariates were included to (1) assess microstructural effects specifically, and (2) to correct for volume differences potentially leading to differences in partial volume effects. Across all regression models, covariates of no interest also included age, sex, interval between baseline and follow-up. As participants were drawn from various waves, we included *number of prior visits* as a covariate in models including memory change to account for potential learning effects. To test whether a relation between sleep and hippocampal MD change was similar across the adult lifespan, we assessed the interaction between the PSQI measure and age. To test for mediation of hippocampal MD change, we performed a mediation analysis across 10,000 bootstrapped samples (R package *mediation* v4.5.0 [68]) [69]. In the Lifebrain consortium data, to test for the relation between sleep and memory change, we calculated partial correlations between sleep and memory change for each sample, correcting for age, sex, and interval between memory tests. We submitted the resulting correlations and corresponding sample sizes to a meta-analysis (R package *meta* v4.9–8 [70]). To illustrate the individual data points, and to provide a general measure of effect size, we extracted hippocampal MD SPC values and the PSQI measure of interest, removed the effects of the nuisance regressors, and plotted the resulting residuals. For the analyses including PGSs, the first three principal components of the genetic ancestry factors were included as covariates to correct for population substructures. To account for potential influences of depression and cognitive impairment, we undertook two sensitivity analyses. First, we tested whether sleep was related to MD hippocampal change when adding depression, both baseline and change scores, to the covariates in the main analysis (scores from BDI (*n* = 172, median [min–max] baseline age = 51 [20–81] years) and GDS (*n* = 43, median [min–max] baseline age = 73 [70–81] years) were entered together, with a separate term controlling for depression scale. Second, we excluded the 11 participants with high depression scores, and the two participants with a low MMS score, and assessed the similarities with the main results.

## Results

### Sleep and age

Summary measures of the PSQI variables can be found in Table 1, together with the correlations between PSQI variables, and between PSQI variables and age. The global score, duration, efficiency, disturbance, and daytime dysfunction, but not quality and latency, showed significant relations with age.

### Microstructural hippocampal change and memory change

**Figure 2.**
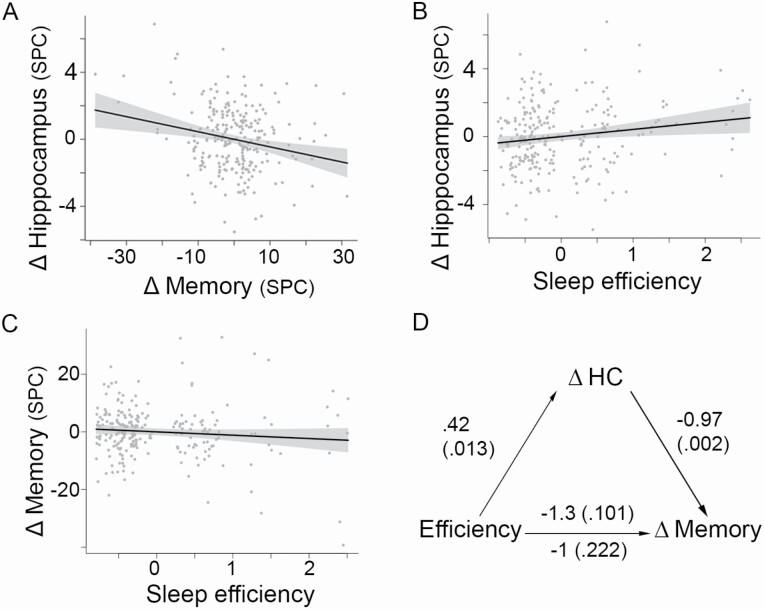
Sleep, and decline in microstructural hippocampal, and memory. (A) Decline in memory related to MD increase in hippocampus (decline in structural integrity). Values are residuals after regressing out covariates (see also Figure 1, B). (B) Sleep efficiency related to hippocampal MD change, independently of hippocampal volume and hippocampal volume change, after FDR-correction for multiple comparisons. (C) Sleep efficiency correlated weakly with memory change (partial *r* = −.11, correcting for age at baseline, interval, sex, and number of prior visits). (D) Average causal mediation effect, that is, the indirect effect of sleep on memory via hippocampus, was −0.41 (*p* =.010).

Figure S3 shows scatterplots for (1) age and hippocampal MD change, using (A) raw values, and (C) adjusted for sex and interval between scans, and (2) age and memory change, using (B) raw values, and (D) adjusted for sex, interval between scans, and number of prior visits. Hippocampal microstructural change related to memory change (*p* < .001, *R*^2^ =.041, Figure 2, A) after accounting for covariates. Higher hippocampal MD change values, interpreted as reduced structural integrity [71], related to more memory decline. Results were similar across the age range (memory change × age interaction term *p* = .661), and when adding baseline IQ to the covariates (memory change *p* < .001).

### Sleep and microstructural hippocampal change.

We found a relation between hippocampal MD change and (1) the global PSQI score (FDR-corrected *p* (*p*_FDR_) < .05, *R*^2^ =.024, Figure S4), uncorrected *p* (*p*_uncorr_) = .012), and (2) sleep efficiency (*p*_FDR_ < 0.05, *R*^2^ =.023, *p*_uncorr_ = 0.013, Figure 2, B). The relations revealed that participants with poorer sleep (higher scores) showed more increase in hippocampal MD, independently of hippocampal volume and hippocampal volume change. The relations did not differ across the age range (*p*_uncorr_ ≥ 0.276). To further assess this lack of sleep × age interactions, we calculated the 95% confidence intervals (CIs) for the regression coefficient of this interaction term across the seven models. The minimum and maximum of the CIs were −0.034 and 0.036, respectively, indicating a maximum decrease or increase in hippocampal MD of ~0.035 SPC for each unit increase in sleep × age variables. We then calculated the expected annual change in hippocampal MD, using a linear model with interval in years between baseline and follow-up MRI as independent variable, correcting for baseline age. This model yielded a regression coefficient of expected annual change of 0.12 (SPC). As 0.036 is one third of 0.12, we are confident that the effect of a unit increase in sleep on aging is less than half the effect of one year of hippocampal MD change. The null findings are also in line with a recent study [8] which found similar sleep-hippocampal atrophy relations across the adult lifespan in a large, longitudinal sample (5,116 scans from 1,299 participants). As the sleep efficiency measure conveys more specific information regarding sleep than the global sleep score, we selected this measure for further analyses.

### Sleep efficiency and memory change

Poor sleep efficiency was not strongly related to memory decline (*p* = .097, *R*^2^ = .011, partial r = −.11, Figure 2, C). To test if this result accurately reflected the true relation, we performed a meta-analysis in five samples from the Lifebrain consortium (*n* = 1,196). This analysis yielded a partial correlation of −.08 (95% confidence intervals (CI) [−0.13, −0.02]), *Z* = −2.70, *p* = .007). The partial correlation obtained in the main sample was within this confidence interval, suggesting a relation between sleep efficiency and memory change may exist, but needing a larger sample to detect it.

### Sleep efficiency, microstructural hippocampal change, and memory change

Although the main sample analysis showed that sleep efficiency was not strongly related to memory decline, we ran a mediation analysis to test for hippocampal MD change as a mediator between sleep efficiency and memory decline (Figure 2, D). Here, we followed emerging perspectives [72–74], arguing that a nonsignificant hypothesis test of the direct efficiency-memory relation does not exclude the potential indirect effects, in this case, via hippocampal decline, which we find to be theoretically plausible pathway [73]. The unstandardized indirect effect on memory change from sleep efficiency via hippocampal MD change was 0.42 × −0.97 = −0.41, similar to the median bootstrapped unstandardized indirect effect of −0.41 (*p* = .019, 95% CI [−0.90, −0.06], *ρ* at which the effect equals 0 was −0.19). The median direct effect estimate, from sleep efficiency to memory change controlling hippocampal MD change, was −1 (*p* = .222). These results suggested that hippocampal MD change partly mediated the relation between sleep efficiency and memory change.

### Sleep efficiency, hippocampal change, and genetic effects

The sleep efficiency PGS did not relate to worse self-reported efficiency (partial *r* = −.04, *p* = .619, Figure S5, A). Lower genetic propensity for efficient sleep related more strongly, but still very modestly, to hippocampal MD change (partial *r* = −.13, *p* = .087, Figure S6, A). For *APOE*, a total of 70 participants carried one or two ε4 alleles. *APOE* ε4 status was not related to sleep efficiency (*r* = −.05, *p* = .526), or hippocampal MD change (*r* = .08, *p* = .307). Re-running the main analysis above adding the sleep efficiency PGS and *APOE* ε4 status, PSQI sleep efficiency still related to hippocampal MD change (*p* = .031).

Higher genetic risk for AD was not related to worse sleep efficiency, that is, higher PSQI scores (partial *r* = .03, *p* = .739, Figure S5, B), or lower hippocampal MD change (partial *r* = −.06, *p* = 0.432, Figure S6, B). Re-running the main model adding the AD PGS and *APOE* ε4 status, sleep efficiency still related to hippocampal MD change (*p* = .023).

### Sleep efficiency, hippocampal change, and Aβ

**Figure 3.**
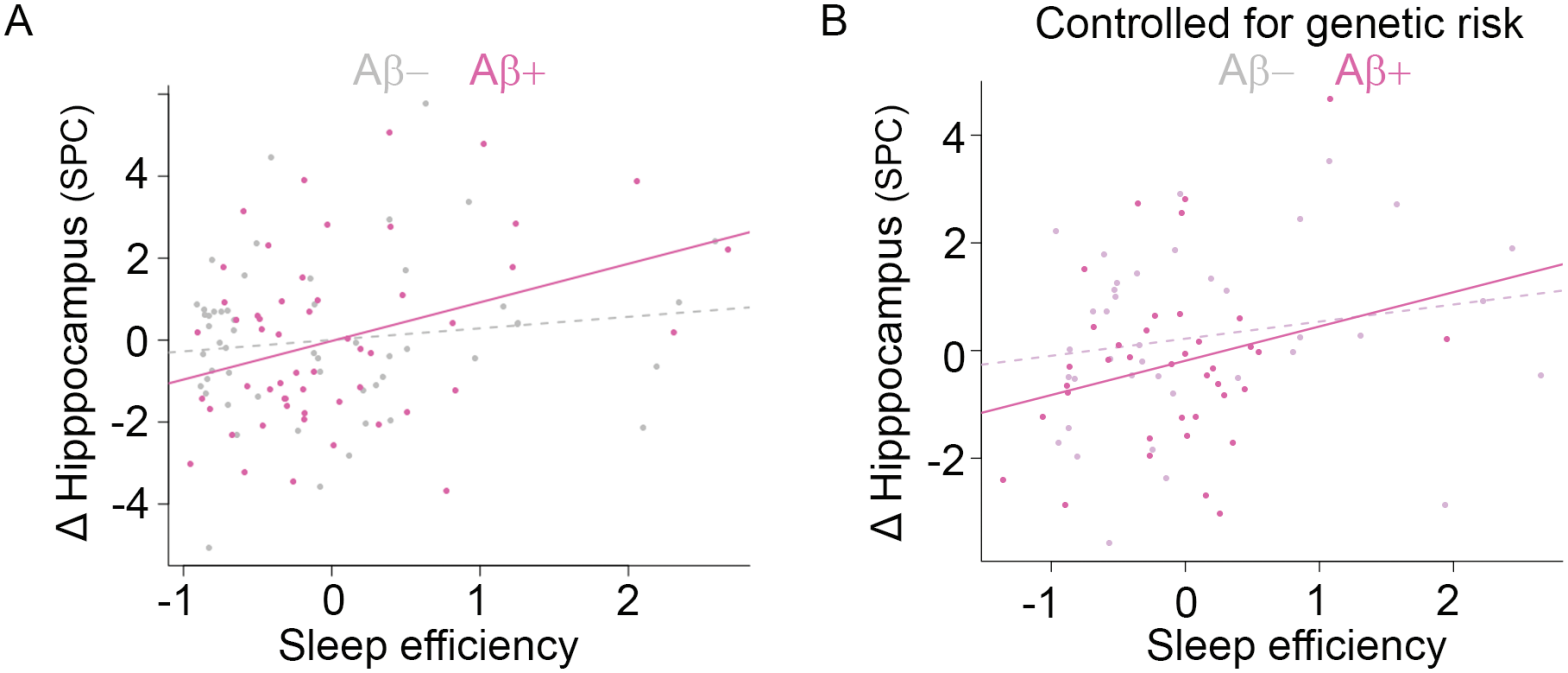
Sleep efficiency, microstructural hippocampal decline, and Aβ accumulation. (A) Efficiency related more strongly to microstructural hippocampal decline in participants with signs of cortical Aβ accumulation. (B) This relation remained when controlling for APOE ε4 status and PGS for sleep efficiency and AD, respectively.

Of 108 participants with PET data, 23 participants were classified as Aβ positive and 85 Aβ negative (Figure S6), with no differences between the two groups in baseline age or MRI interval (*p* = .215 and .383, respectively). We found a stronger relation between sleep efficiency and hippocampal MD change in participants classified as Aβ positive (efficiency × Aβ interaction term *p* = .022, Figure 3, A). Including baseline IQ yielded a similar result (interaction term *p* = .017). Comparing Aβ negative and Aβ positive participants did not show differences in sleep efficiency (*p* = .729), hippocampal MD decline (*p* = .932), or memory decline (*p* = .680). When repeating the analysis in the Aβ positive and negative groups separately, we observed a relation between sleep efficiency and hippocampal MD change only in the Aβ positive (*p* = .019), but not in the Aβ negative subgroup (*n* = 85, *p* = .361).

### Sleep efficiency, hippocampal change, Aβ, and genetic effects

A subsample of 76 participants (mean (*SD*) age = 69.3 (8.2) years, min−max 44–81 years) had both Aβ and genotype data, and 24 of these participants had one or two *APOE* ε4 alleles. We included the *APOE* ε4 status and the PGS for sleep efficiency and AD, respectively, to the initial model. The results demonstrated (1) that sleep efficiency still related to hippocampal MD change differently for Aβ negative and positive participants (*p* = .015, Figure 3, B), and (2) an effect of the PGS for sleep efficiency on hippocampal MD change (*p* = .028), with higher propensity of efficient sleep showing less MD hippocampal decline. The AD PGS showed a very weak effect (*p* = .087), while the *APOE* genotype showed no effect (*p* = .537).

### Sensitivity analyses

We verified that (1) when controlling for levels of depression (both baseline and change scores), sleep efficiency still related to MD hippocampal change (sleep efficiency *p* = .004), and (2) when excluding the participants with depression and MMS scores beyond threshold values, the results remained highly similar. That is, sleep efficiency related to hippocampal MD change (*p* = .011), as in the main analysis, and weakly to memory change (partial *r* = −.09, similar to the *r* values found in the main analysis and in the meta-analysis), while the relation between sleep efficiency and hippocampal MD change differed depending on cortical Aβ accumulation (*p* = .012).

## Discussion

The results indicate that sleep efficiency and hippocampal microstructural decline are related in presence of cortical Aβ accumulation. This relation does not appear to be explained by *APOE* ε4 genotype, or polygenic scores for sleep efficiency or AD. Sleep efficiency related to memory reduction indirectly via the intra-hippocampal integrity decline. Although we cannot rule out that these Aβ-related associations stem from unexplored factors such as tau deposition, Aβ accumulation might make people more vulnerable to the effects of hippocampal integrity on sleep, or vice versa, leading to decline in episodic memory.

As the observed hippocampal effects were independent of baseline hippocampal volume and volume change, microstructural change in the hippocampus might be a particularly sensitive marker of early decline, complementary to atrophy. In support of this hypothesis, two previous studies of 147 (overlapping with the current sample) [26] and 66 [75] participants, respectively, did not observe relations between sleep and hippocampal volume or atrophy, while one cross-sectional study including 1,201 young adults reported associations between right hippocampal MD and sleep quality (but not sleep duration) [76]. These findings also suggest larger samples are needed to detect the sleep-atrophy relations. In support of this notion, in 3,105 cognitively normal participants aged 18–90 years, including participants from the present sample, we found that poorer sleep efficiency, as well as sleep quality, problems, and daytime tiredness, were related to greater hippocampal volume loss [65]. Relations might also surface given a longer follow-up interval, as increased thinning was found in 122 older adults with either short (<7 h) or long (>7 h) sleep compared with intermediate (7 h) sleep duration, over a mean interval of 8 years [24]. The current finding supports these relations between self-reported sleep and brain change but extends previous knowledge by revealing independent intra-hippocampal reductions in microstructural integrity.

The mechanisms of microstructural hippocampal decline remain unclear, but may relate to decay of the dendritic architecture. In mice, hippocampal dendritic spine densities have been shown to be reduced in aging [77], and after sleep deprivation [10], with reductions relating to memory defects [78]. Over time, reduction of spines and synapses might promote larger dendritic disruptions, detected in mice via intra-hippocampal DTI, and linked to memory impairments [79]. In humans, these speculations could be tested using ultrahigh-resolution DTI [80].

The relation between sleep and Aβ appears reciprocal, as Aβ accumulate after sleep deprivation [81], and increases wakefulness and alters sleep patterns [82]. Here, although Aβ status did not relate to sleep efficiency (in contrast to [2], and a study [83], which found relations with sleep duration and quality) or hippocampus decline alone (as in 84, but see [85]), the sleep-hippocampal decline relation was stronger in the Aβ positive. Echoing this finding, in a separate sample of older adults, we recently observed that tau and YKL-40, a biomarker of inflammation and astroglial activation, related more strongly to the PSQI global score in Aβ positive than in Aβ negative [86]. These results raise the possibility that sleep problems signal Aβ accumulation co-occurring with other adverse signs such as inflammation or hippocampal decline.

As we observed a relation between sleep efficiency and memory decline mediated by higher hippocampal diffusivity, we hypothesize that hippocampal decline, when concomitant with cortical Aβ accumulation, causes sleep problems, here in the form of poorer sleep efficiency, and memory decay. A candidate mechanism could be subtle alterations of sleep-related cortico-hippocampal coupling. For instance, during sleep, slow waves (as seen in the electroencephalograph) propagate from the cortex to hippocampus [87], and bilateral hippocampal damage was recently shown to have a substantial effect on such cortical oscillations [7]. The resulting poorer sleep could further interfere with hippocampal-dependent memory processes [88].

The current data does not allow inferences that rule out the reverse causality, of sleep affecting hippocampal decline, potentially partly via reduced glymphatic clearance of potentially neurotoxic substrates which might predispose to Aß and tau pathology [89] However, the effects here were specific for sleep efficiency, rather than the sleep duration or sleep quality components of the PSQI, both potentially more likely drivers of such potential sleep-generated effects. Likewise, we cannot rule out that a variable not assessed here can account for the observed associations [69]. For instance, sleep spindles have been linked to both Aβ and tau [3], and tau potentially related to AD is first detected in the locus coeruleus [90]. Activity in this region can alter sleep spindles, affecting memory consolidation [91]. As subjective sleep reports were used here, to tease out causal pathways, studies could probe sleep with physiological measurements and follow Aβ-negative participants with healthy sleep patterns and no signs of hippocampal decline, to detect changes in sleep patterns, hippocampal integrity, Aβ, tau, memory, and neuroinflammation markers like YKL-40 or sTREM2. Intervention studies targeting for instance hippocampal-dependent cognition [92], and investigating similar markers could be a less costly strategy.

The associations remained after controlling for genetics risk indexed by PGSs for sleep efficiency and AD, respectively, as well as the presence of the *APOE* ε4 allele. The latter finding was in agreement with previous findings [23, 24]. For sleep PGS, in ~7,000 participants, a relation has been reported between self-reported sleep duration and PGS for sleep duration [93]. No relation was found between hippocampal atrophy in 421 cognitively healthy, older adults and AD genes from an exploratory GWAS [94]. For both *APOE* and the PGSs, we observed weak relations with sleep efficiency and hippocampal diffusivity change, respectively. Such associations must be resolved in larger samples before we can draw the conclusion that the relation between sleep efficiency and hippocampal decline is partly independent of genetics.

Limitations of this study include the use of a self-report measure of sleep, at one time point, not necessarily in the same month as baseline MRI, instead of objective measures such as activity monitors, or polysomnography, collected repeatedly, starting within a month of the baseline MRI. Although self-reported sleep measures might provide more representative data on sleep than a single-night polysomnography [95], a relatively modest correlation of .47 has been reported between reported and measured sleep duration [96]. In future studies, a likely key is repeated measurements of sleep patterns, assessment of potential underlying sleep disorders, and the inclusion of other biomarkers. Inclusion of such markers would also improve analyses of mediation, which here does not establish causality. As the sleep efficiency PGSs stem from a GWAS using activity monitors [45], the inclusion of objective sleep measures could shed further light on the relative contribution of sleep genetics and sleep behavior. Although the current sample is relatively large, the potentially complex interplay between Aβ positivity and other markers of relatively low prevalence highlights the need for even larger sample sizes.

The results indicate that hippocampal microstructural decline related to sleep efficiency in Aβ positive participants, and mediated the link between sleep and episodic memory change across the adult lifespan. This relation was not readily explained by genetic effects. Poor self-reported sleep efficiency might constitute a separate risk marker for AD, and future studies need to address why sleep is related to more hippocampal decline in Aβ positive older adults even without dementia.

## Supplementary Material

zsab110_suppl_Supplementary_Materials
