# Supplementary material for: Self-reported sleep relates to microstructural hippocampal decline in ß-amyloid positive Adults beyond genetic risk
Source: Sleep. 2021 Apr 28;44(11):zsab110. doi: 10.1093/sleep/zsab110 (PMC8598196; doi:10.1093/sleep/zsab110)
Supplement: zsab110_suppl_Supplementary_Materials [file zsab110_suppl_supplementary_materials.docx]

Supplemental Information for:

**Self-Reported Sleep Relates to Microstructural Hippocampal Decline in β-Amyloid Positive Adults Beyond Genetic Risk**

Håkon Grydeland^1^, Donatas Sederevičius^1^, Yunpeng Wang^1^, David Bartrés-Faz^3^, Lars Bertram^1,4^, Valerija Dobricic^4^, Sandra Düzel^5^, Klaus P. Ebmeier^6^, Ulman Lindenberger^5,7^, Lars Nyberg^1,8^, Sara Pudas^8^, Claire E. Sexton^6^, Cristina Solé-Padullés^3^, Øystein Sørensen^1^, Kristine B. Walhovd^1, 2^, Anders M. Fjell^1,2^

Research Group for Lifespan Changes in Brain and Cognition, Department of Psychology, University of Oslo (H.G., K.B.W, Y.W., D.S., A.M.F)

Department of Radiology and Nuclear Medicine, University of Oslo (K.B.W, A.M.F)

Departament de Medicina, Facultat de Medicina i Ciències de la Salut, Universitat de Barcelona, Spain (D.B.-F., C.S.-P.)

Max Planck Institute for Molecular Genetics, Germany (L.B.)

Max Planck Institute for Human Development, Germany (S.D., U.L.)

Department of Psychiatry, University of Oxford, UK (K.P.E., C.E.S.)

Umeå Center for Functional Brain Imaging, Umeå University, Sweden (L.N., S.P.)

Corresponding author: Håkon Grydeland, Department of Psychology, University of Oslo, PO Box 1094 Blindern, 0317 OSLO, Norway, telephone: +47 22845067, email: [hakon.grydeland@psykologi.uio.no](mailto:hakon.grydeland@psykologi.uio.no)

Contents

[Supplementary Figures 3](#_Toc66707058)

[**Figure S1**. Attrition. 3](#_Toc66707059)

[**Figure S2**. Age distribution at baseline (n=243). 4](#_Toc66707060)

[**Figure S3**. Age relations with hippocampal and memory change. 5](#_Toc66707061)

[**Figure S4**. Global sleep quality related to hippocampal MD change. 6](#_Toc66707062)

[**Figure S5**. Self-reported sleep efficiency and polygenic scores 7](#_Toc66707063)

[**Figure S6**. Hippocampal MD change and polygenic scores 8](#_Toc66707064)

[**Figure S7**. Distribution and classification of Aβ. 9](#_Toc66707065)

# Supplementary Figures

## **Figure S1**. Attrition.

**
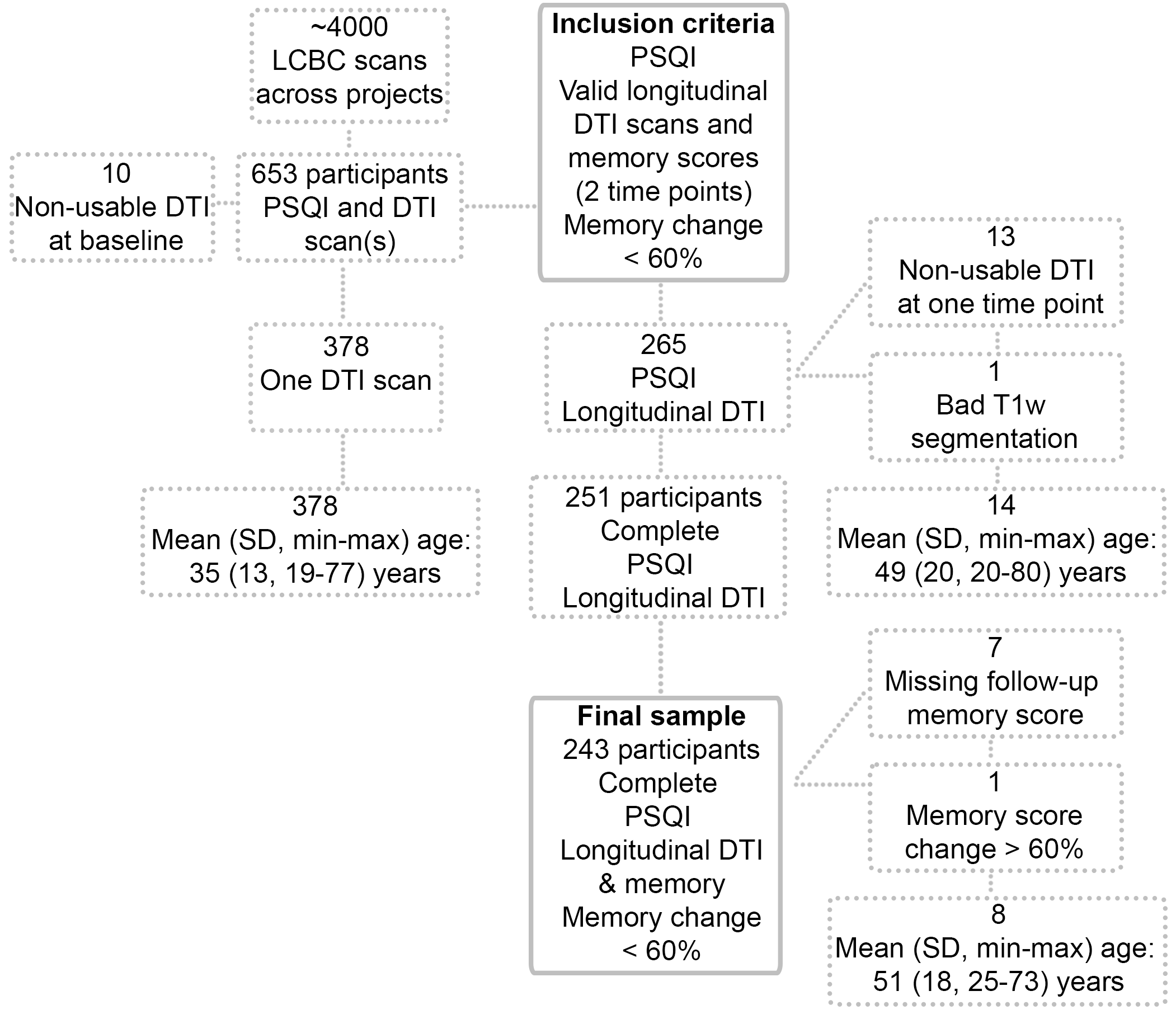
**

## **Figure S2**. Age distribution at baseline (n=243).

## **Figure S3**. Age relations with hippocampal and memory change.

Relations between (i) age and hippocampal MD change, using (A) raw values, and (C) adjusted for sex and interval between scans (R^2^=0.056, p<0.001), and (ii) age and memory change, using (B) raw values, and (D) adjusted for sex, interval between scans, and number of prior visits (R^2^=0.028, p=0.009).


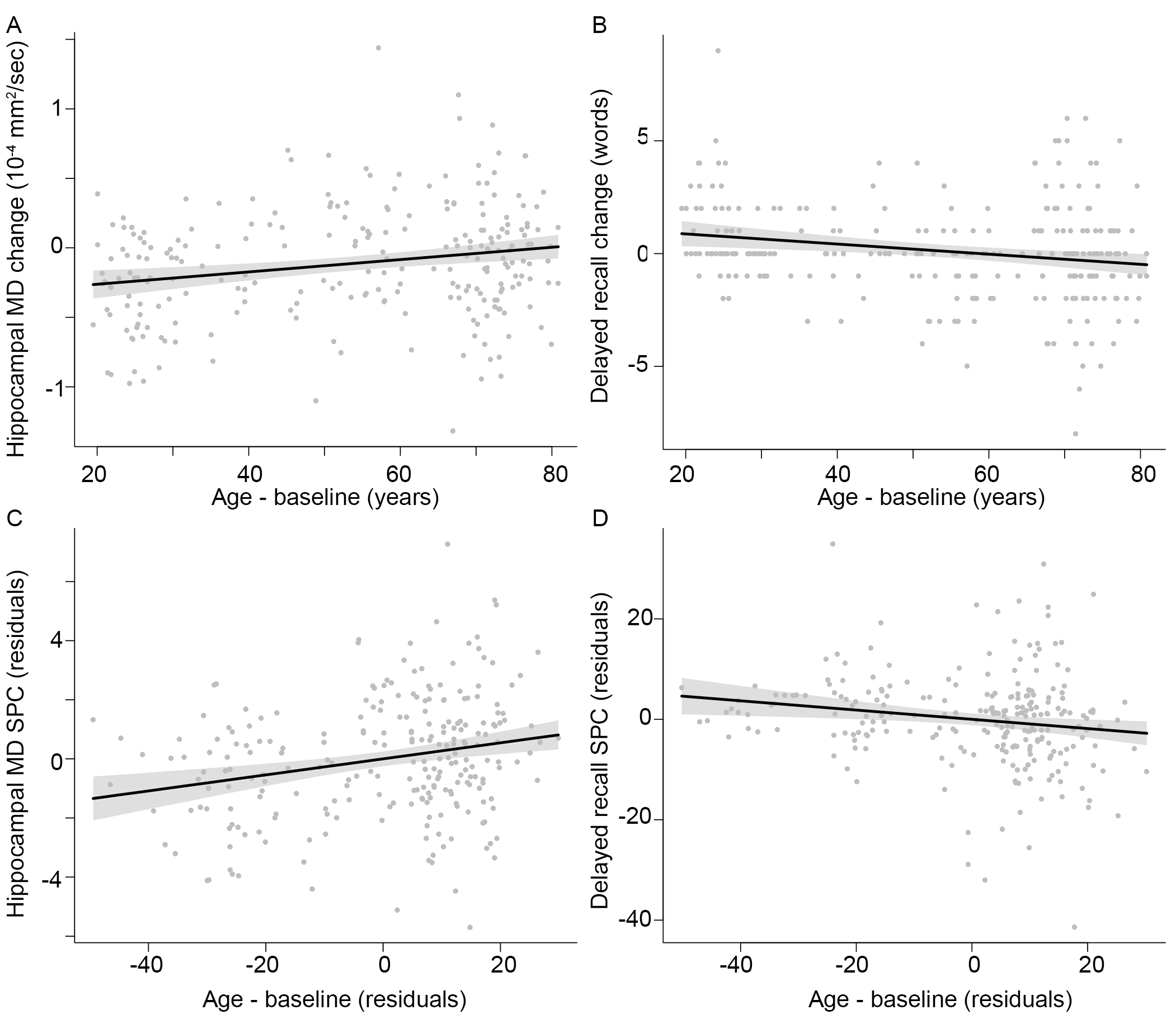


## **Figure S4**. Global sleep quality related to hippocampal MD change.


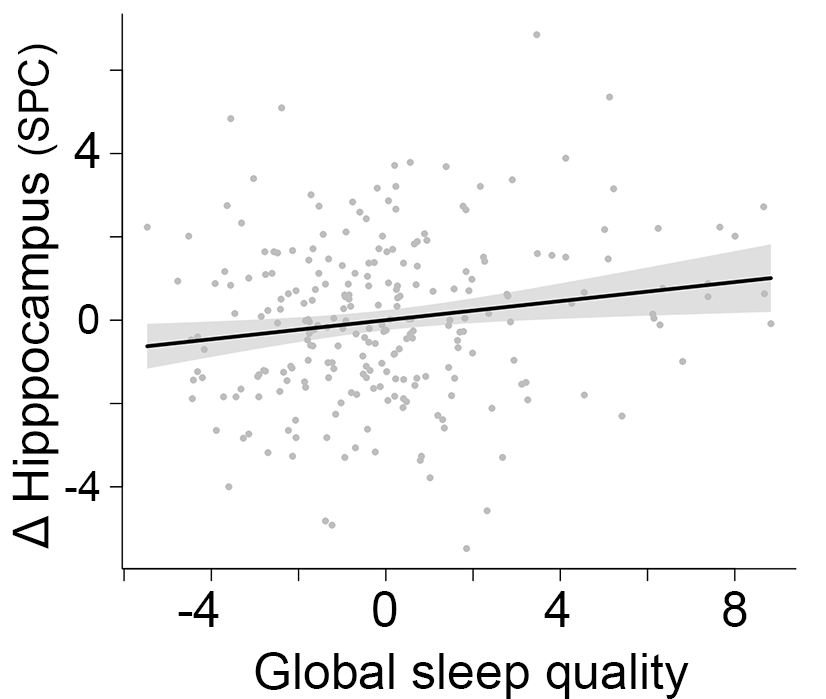


## **Figure S5**. Self-reported sleep efficiency and polygenic scores

Self-reported sleep efficiency and polygenic scores (PGSs) for (A) sleep efficiency, and (B) AD.


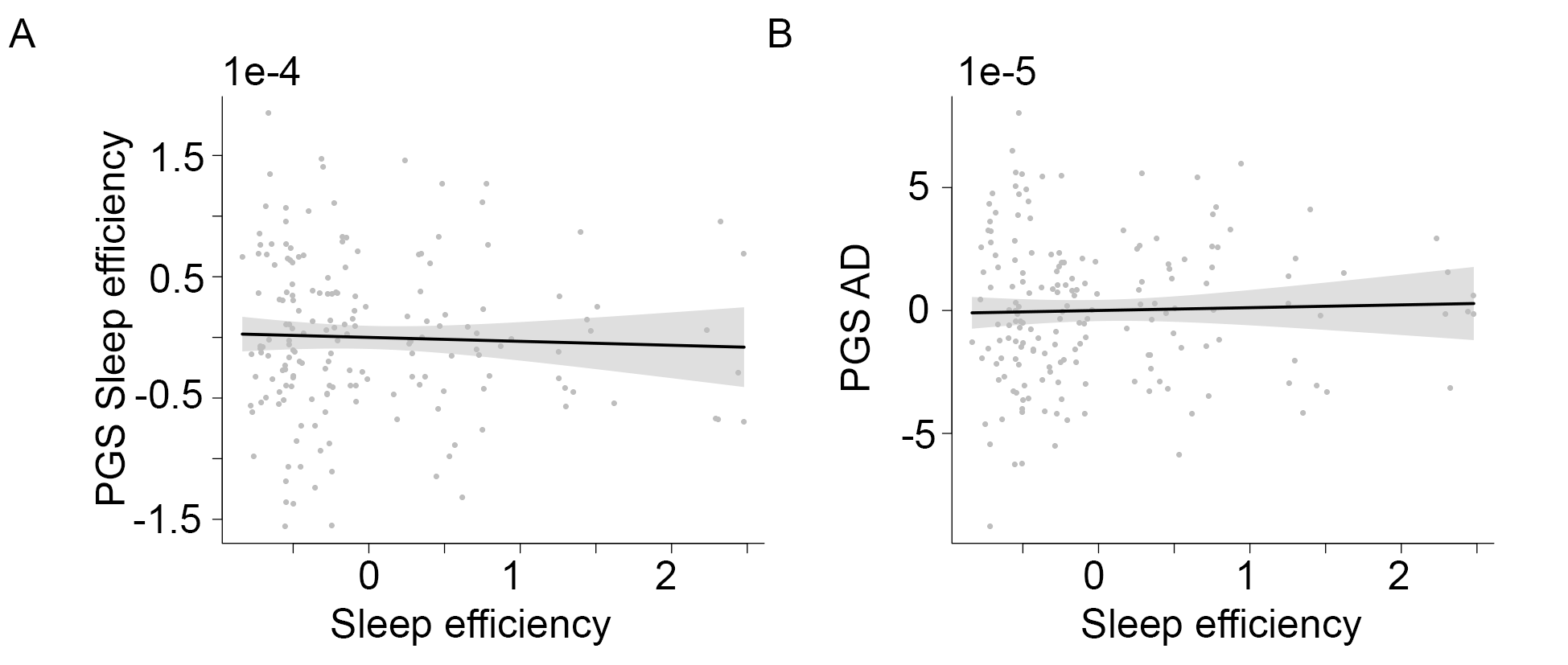


## **Figure S6**. Hippocampal MD change and polygenic scores

Hippocampal MD change and polygenic scores (PGSs) for (A) sleep efficiency, and (B) AD.


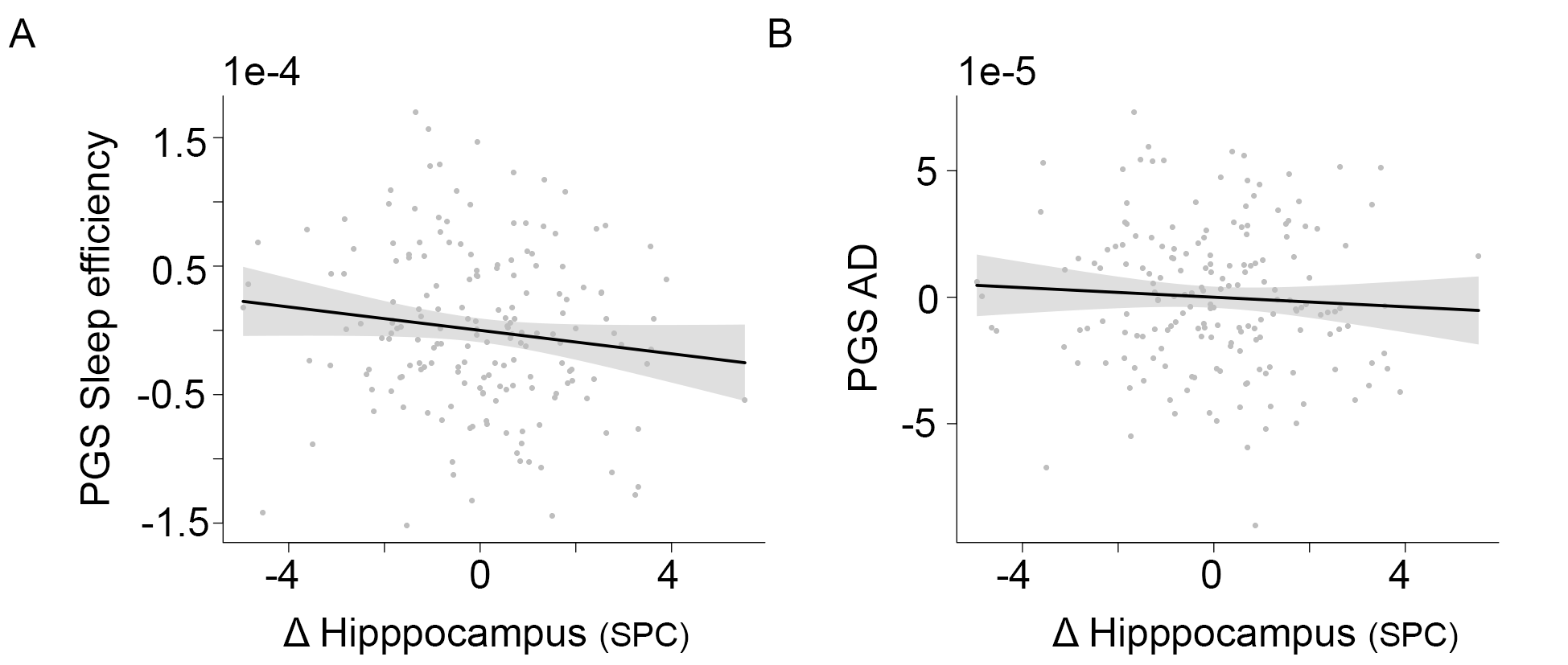


## **Figure S7**. Distribution and classification of Aβ.


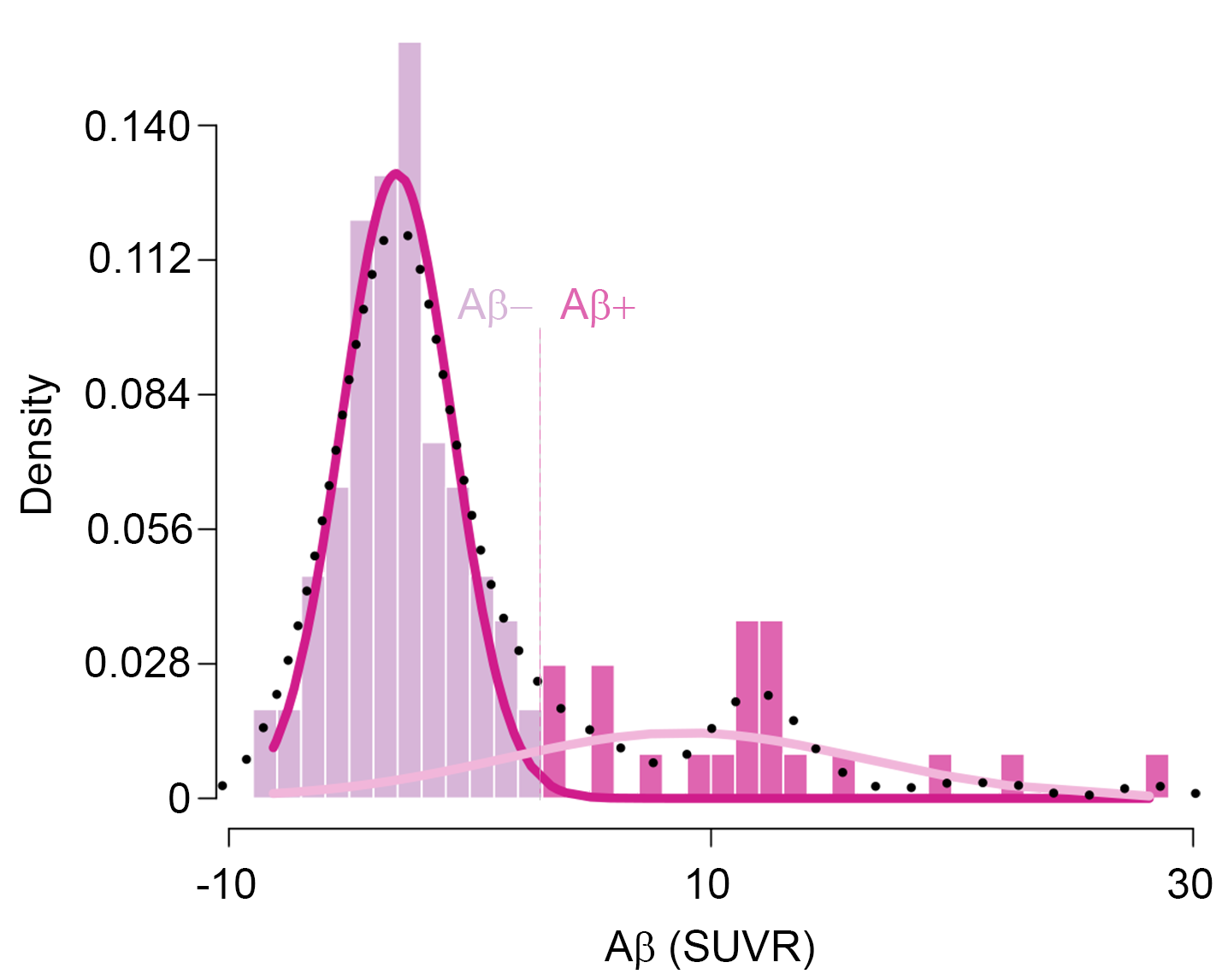


The best fit was a two-distribution solution (with unequal variance), represented in different colours. Fit of the distributions overlaid together with actual density (black dotted line). SUVR=standardized uptake value ratios. Aβ negative sample: n=85 (60%F), mean (SD, min-max) age: 67.4 (9.1, 44.4-80.8) years. Aβ positive sample: n=23 (48%F), mean (SD, min-max) age: 70 (6.6, 51.1-78.6) years.
